# Clinical-grade human umbilical cord-derived mesenchymal stem cells reverse cognitive aging via improving synaptic plasticity and endogenous neurogenesis

**DOI:** 10.1038/cddis.2017.316

**Published:** 2017-08-10

**Authors:** Ning Cao, Tuling Liao, Jiajing Liu, Zeng Fan, Quan Zeng, Junnian Zhou, Haiyun Pei, Jiafei Xi, Lijuan He, Lin Chen, Xue Nan, Yali Jia, Wen Yue, Xuetao Pei

**Affiliations:** 1 Stem Cell and Regenerative Medicine Lab, Beijing Institute of Transfusion Medicine, Beijing, China; 2 South China Research Center for Stem Cell and Regenerative Medicine, South China Institute Biomedicine, Guangzhou, China

## Abstract

Cognitive aging is a leading public health concern with the increasing aging population, but there is still lack of specific interventions directed against it. Recent studies have shown that cognitive function is intimately affected by systemic milieu in aging brain, and improvement of systemic environment in aging brain may be a promising approach for rejuvenating cognitive aging. Here, we sought to study the intervention effects of clinical-grade human umbilical cord-derived mesenchymal stem cells (hUC-MSCs) on cognitive aging in a murine model of aging. The conventional aging model in mice induced by d-galactose (d-gal) was employed here. Mice received once every two weeks intraperitoneal administration of hUC-MSCs. After 3 months of systematical regulation of hUC-MSCs, the hippocampal-dependent learning and memory ability was effectively improved in aged mice, and the synaptic plasticity was remarkably enhanced in CA1 area of the aged hippocampus; moreover, the neurobiological substrates that could impact on the function of hippocampal circuits were recovered in the aged hippocampus reflecting in: dendritic spine density enhanced, neural sheath and cytoskeleton restored, and postsynaptic density area increased. In addition, the activation of the endogenic neurogenesis which is beneficial to stabilize the neural network in hippocampus was observed after hUC-MSCs transplantation. Furthermore, we demonstrated that beneficial effects of systematical regulation of hUC-MSCs could be mediated by activation of mitogen-activated protein kinase (MAPK)-ERK-CREB signaling pathway in the aged hippocampus. Our study provides the first evidence that hUC-MSCs, which have the capacity of systematically regulating the aging brain, may be a potential intervention for cognitive aging.

Cognitive aging is a lifelong process of ongoing and gradual cognitive function decline in the senior. Its physiological characteristics are authenticated that large neurons appear to shrink, few are lost, but its neurobiological substrates for function are reduced neuroplasticity and neurogenic potential.^1, 2^ Although cognitive aging is not considered as a disease, it affects daily life of older adults and their families and brings significant social pressure.^1, 3^ How to maintain cognitive integrity and prevent further deterioration of cognition have emerged as a leading public health concern with the increasing aging population,^4^ but there is still lack of specific interventions.

It has gradually been recognized that the aging systemic milieu negatively regulates cognitive function in aging brain, reflecting in impaired spatial learning and memory, decreased synaptic plasticity and neurogenesis and so on. In the young brain, the local microenvironment is vital for stable neural structure and function and maintaining normal neurogenesis.^5, 6^ Thus, positive regulation of systemic environment in aging brain might be particularly effective, and corresponding systemic strategies might hold great promise for the restoration of aging conditions.^5, 6, 7^

Human umbilical cord-derived mesenchymal stem cells (hUC-MSCs), are closer to the fetal phase, easier to collection, and have higher proliferation and faster self-renewal ability compared with MSCs from other sources such as bone marrow, adipose tissue.^8, 9, 10^ More importantly, hUC-MSCs can secrete a wide range of functional factors, including growth factors, cytokines, chemokines and metabolites, which are very important to regulate multiple physiological functions of the aged organism.^10^ Whether transplantation of hUC-MSCs could systematically regulate the aged brain and alleviate cognitive aging is still unclear. Therefore, we hypothesized that hUC-MSCs, as young stem cells,^11^ may be a superior source for reversing cognitive aging by providing circulating multifunctional factors and improve systemic environment.

Here, for the first time, we explored the effects of clinical-grade hUC-MSCs on recovery of cognitive aging. We selected a d-galactose (d-gal)-induced aging model, a systemic and homogeneous aging model with the acceleration of aging and cognitive deficits.^12, 13, 14^ To observe the systematic regulation effects in aging brain by the secreted multifunctional factors from hUC-MSCs, hUC-MSCs were infused into the d-gal-induced aging mice intraperitoneally. We found that administration of hUC-MSCs could upregulate plasticity-related genes, reverse the spine loss and promote synaptic plasticity in the aged hippocampus. We further demonstrated that hUC-MSCs promote the endogenic neurogenesis and stabilize the neural network in hippocampus. Mechanistically, the structural recovery and cognitive enhancements elicited by exposure to the multifunctional factors secreted from hUC-MSCs were at least partially mediated by activation of the cyclic AMP response element binding protein (CREB) in the aged hippocampus through the activation of MAPK-ERK signaling pathway. Collectively, our study provides a promising strategy to prevent cognitive aging by systemic factors secreted by hUC-MSCs.

## Results

### Optimizing the methods for the isolation and culture of clinical-grade hUC-MSCs

**Figure 1:**
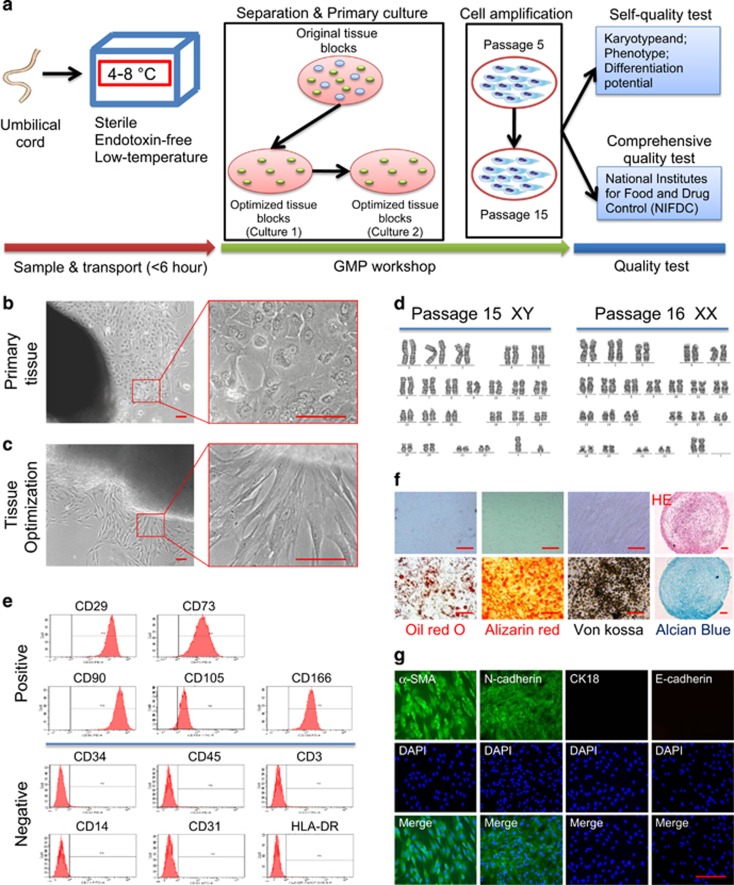
Optimization of isolation and culture for clinical-grade hUC-MSCs. (**a**) Schematic of preparation and quality test for clinical-grade hUC-MSCs. (**b**) Representative tissue block with non hUC-MSCs in the primary culture of the original umbilical cord tissue blocks. (**c**) Representative of optimized tissue blocks with good clones. (**d–g**) Several representative characteristics of optimized tissue blocks-derived hUC-MSCs, including phenotype, karyotype and differentiation potential. (**d**) Representative karyotype analysis of hUC-MSCs derived from different genders. hUC-MSCs amplified from the reused optimized tissue blocks both had the normal karyotype (Left: Passage 15, a normal male karyotype; Right: Passage 16, a normal female karyotype). (**e**) Flow cytometric analysis showed hUC-MSCs were positive for mesenchymal lineage markers (CD29, CD73, CD90, CD105 and CD166), negative for hematopoietic and endothelial markers (CD34, CD45, CD3, CD14 and CD31), and negative for HLA-DR. (**f**) Differentiation potential of hUC-MSCs into mesodermal lineages. Representative images of hUC-MSCs differentiated into adipocytes, osteocytes and chondrocytes are shown as indicated. Fat droplets were stained with Oil red O. Calcium phosphate deposits were stained with Alizarin Red, and lots of calcium deposition in the extracellular matrix, which were verified by von Kossa staining. Proteoglycans with Alcian Blue. (**g**) Immunofluorescence staining of hUC-MSCs showed they were positive for mesenchymal markers of *α*-SMA (Green) and N-cadherin (Green), and negative for epithelial markers of CK18 and E-cadherin (Scale bar, 200 *μ*m)

Umbilical cord was transported in the sterile, endotoxin-free and low-temperature condition. The time of sampling and transporting was controlled within 6 h before the separation operation (Figure 1a). The whole process included separation, culture, amplification and cryopreservation, was operating in the good manufacturing practice (GMP) workshop. In order to obtain the high-quality stem cells, we used modified tissue block cultivation method with independent intellectual property right and patent (Patent application no. 201510459332.0). After the first adherent culture, the original umbilical cord tissue blocks were screened with good clones and without endothelial cells. The screened tissue blocks were simply treated with trypsin (37 °C for 5 min) and washed with physiological saline, which could remove the residual cells; then the processed tissue blocks continued to spread evenly on the surface of the culture dish, and a large number of stem cell clones with high purity and better statue would produce in a shorter period of time. In this way, such high-quality tissue blocks can be reused twice to fully obtain primary cells (Figures 1a–c). We expanded the primary cells to Passage 5 (P5) and Passage 15 (P15), which were then sent to the National Institutes for Food and Drug Control (NIFDC) for comprehensive quality test. By the way of self-quality test, we also performed several representative characteristics of hUC-MSCs including phenotype, karyotype and differentiation potential (Figures 1d–g). All indicators of the both stem cell passages were conformed to the quality standards (Table 1; Supplementary Table 1).

### hUC-MSCs improve spatial learning ability and memory in d-galactose induced aging mice

**Figure 2:**
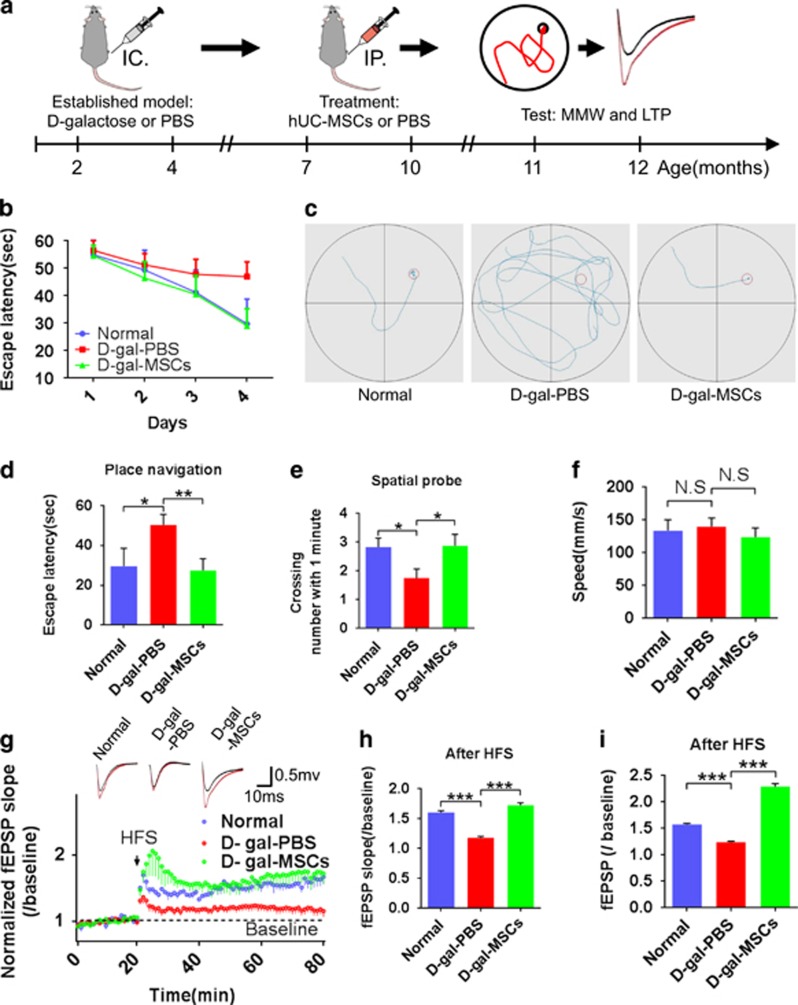
hUC-MSCs improved spatial learning and memory ability and enhanced hippocampal synaptic plasticity in aging mice. (**a**) Schematic illustrating the chronological order used for d-galactose injection, hUC-MSCs treatment, Morris water maze and LTP testing. (**b–f**) Results from Normal, d-gal-PBS and d-gal-MSCs groups that were cognitively tested by Morris water maze (*n*=10–12 mice/group). (**b**) Learning curves show mean daily escape latencies. (**c**) Typical escape way in hidden platform test. (**d**) Escape latencies in day 4. (**e**) Average number of platform crossings (swims over former platform location). (**f**) The swimming speed in day 4. (**g–i**) hUC-MSCs rescued LTP impairment in d-galactose induced aging mice. LTP in the hippocampal CA1 region was induced by high-frequency stimulation (HFS) (*n*=4–7/group). (**g**) Averaged slopes of baseline normalized fEPSP. (Inset) Examples of fEPSP recorded at 5 min before (black) and 55 min after (red) LTP induction. (**h**) Quantification of mean fEPSP slopes during the last 10 min of the recording after LTP induction. (**i**) Quantification of mean fEPSP amplitude during the last 10 min of the recording after LTP induction. (All data shown as mean±S.E.M., *P<0.05, **P<0.01.)

Three month after transplantation of hUC-MSCs, animals were tested on Morris water maze (MWM) task for spatial learning and memory ability. In the first stage, escape latency of d-gal-PBS group was much longer than normal and d-gal-MSCs groups on day 3 and 4 (Figure 2b), especially, on day 4 (Figure 2d). The typical escape way of normal and d-gal-MSCs groups showed immediate and orientated (Figure 2c). Meanwhile, the swimming speed had no obvious differences among the three groups (Figure 2f). In the second stage, after the hidden platform removed, the number of times mice crossing the original platform location within 1 min was counted as an index of the spatial memory of the platform. The times of the d-gal-PBS group was significantly lower than the value obtained in the normal and d-gal-MSCs groups (Figure 2e). These data indicate that transplantation of hUC-MSCs improves cognitive function in aging mice compared with PBS-treated aging animals.

### hUC-MSCs enhance the hippocampal synaptic plasticity long-term potentiation in aging model

Hippocampal synaptic plasticity long-term potentiation (LTP) is thought to be the molecular event that contributes to learning and memory.^15^ After WWM testing, LTP was measured at the Schaffer collateral (SC)-CA1 synapses in hippocampal slices and the field excitatory postsynaptic potentials (fEPSP) from each group were recorded to examine the effects of hUC-MSCs transplantation on hippocampal synaptic plasticity (Figures 2g–i). High-frequency stimulation (HFS) triggered a significant increase in the magnitude of LTP at the SC–CA1 synapses and maintained over 60 min (Figure 2g). d-gal-PBS group exhibited a lower slope of fEPSP than the normal and d-gal-MSCs groups (Figures 2g and h). LTP in the d-gal-PBS group quickly reached baseline levels, however LTP in the normal and d-gal-MSCs groups were maintained above baseline throughout the recording period (Figure 2i). These functional data indicate that synaptic plasticity of aging mice is significantly enhanced by hUC-MSCs.

### hUC-MSCs restore the synaptic structures and physiological state of the neuron in the hippocampus

**Figure 3:**
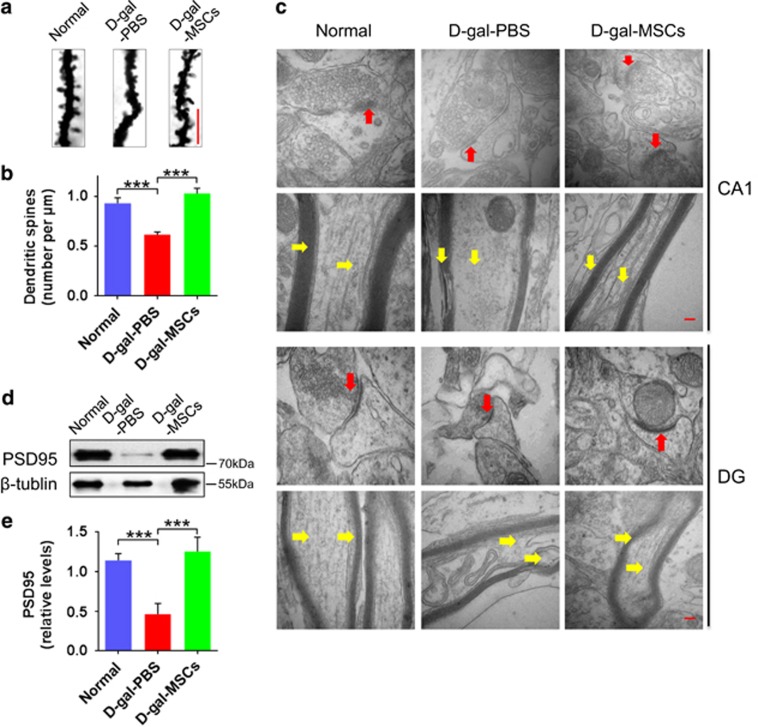
hUC-MSCs increased spine density and recovered synapse ultrastructure in the aged hippocampus. (**a**) Representative Golgi stain image and (**b**) quantification of dendritic spine density on tertiary branches (*n*=5 neurons/3 mice/group, scale bar, 5 *μ*m). (**c**) Typical utrastructural about the microtubules, neurofilament and postsynaptic density (PSD) area of the asymmetrical synapses, in the CA1 and DG area. It can be rejuvenated in d-gal-MSCs mice (scale bar, 100 nm). (**d**) Representative images and (**e**) quantification of western blotting showing the expression of PSD95 in the hippocampal of normal, d-gal-PBS and d-gal-MSCs mice (*n*=4). (All data are shown as the mean±S.E.M., ***P<0.001.)

To clarify the synaptic plasticity changes, we further investigated the hippocampal neuronal structure and physiological state in the mice. Representative Golgi-stained dendritic spines of pyramidal cells in the CA1 region were shown for each group (Figure 3a). The number of dendritic spines in the d-gal-PBS group significantly decreased compared with that of the normal group, while the dendritic spines were reversed in the d-gal-MSCs group (Figure 3b). Previous results have suggested that asymmetrical synapses involve in synaptic plasticity associated with LTP,^16^ especially mushroom spines provide structural storage sites for long-term associative memory and sites for memory-specific synaptogenesis.^17^ To observe their ultrastructure, we examined CA1 pyramidal cells in the middle third of the stratum radiatum (Figure 3c) and granule cells in dentate gyrus (DG) (Figure 3c) by electron microscopy and the results showed that postsynaptic density (PSD) area decreased in the asymmetrical synapses (mushroom spines) of d-gal-PBS mice. Furthermore, we found that PSD95, which can promote synapse maturation and enhance synaptic plasticity,^18^ was markedly upregulated in the hippocampus of d-gal-MSCs group (Figures 3d and e). Meanwhile, in the normal and d-gal-MSCs group, the neural sheath, neuromicrotubes and neurofilaments in the CA1 and DG regions were lined up normally, densely and in order; however, the dissolution and disruption of the neural sheath, disassembly of the neuromicrotubules and neurofilaments (Figure 3c). In addition, we found that the morphology of DG neurons of three groups had no significant differences (Supplementary Figure 1A); whereas the density of nissl bodies (a useful marker for the physiological state of the neuron) significantly decreased in the CA1 and DG regions in the d-gal-PBS group (Supplementary Figure 1B). These data demonstrate that hUC-MSCs restore the dendritic spine loss, and maintaine neural ultrastructure in synaptic, neural sheath, neuromicrotubes and neurofilaments in the aged hippocampus.

### hUC-MSCs rejuvenate endogenic neurogenesis in the aged brain

**Figure 4:**
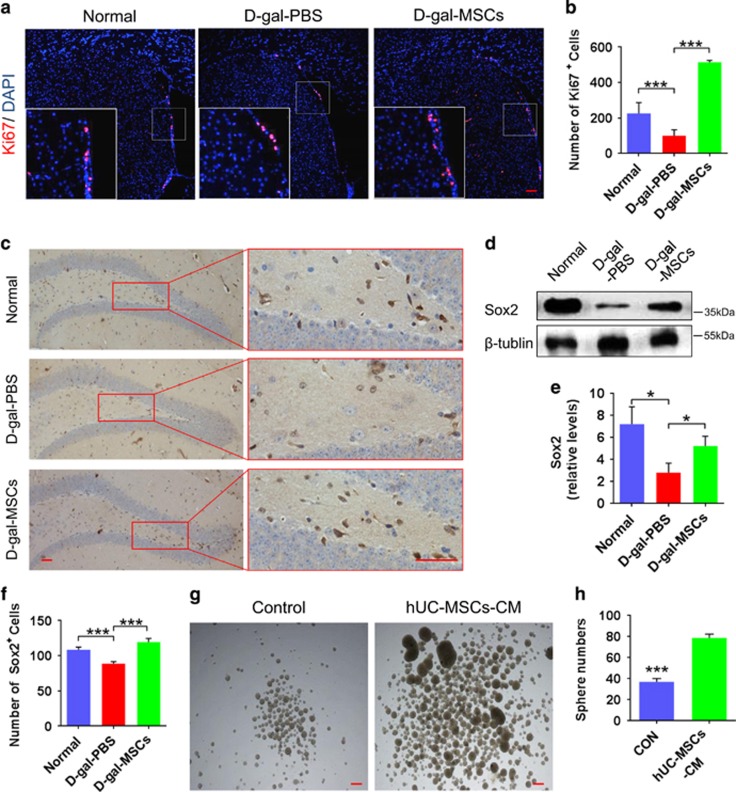
Rejuvenation of endogenic neural progenitor cells by hUC-MSCs. (**a**,**c**) Images showing the effects of hUC-MSCs on (**a**) proliferative cells in the SVZ and (**c**) neural stem cells in the DG of Normal, d-gal-PBS and d-gal-MSCs mice. (**b**,**f**) Quantification of (**b**) proliferative and (**f**) neural stem cell populations of the above images (*n*=7). (**d**) Representative images and (**e**) quantification of western blotting showing the expression of SOX2 in the hippocampal of normal, d-gal-PBS and d-gal-MSCs mice (*n*=4). (**g**) In the culture system, hUC-MSCs condition medium (CM) significantly promoted neural sphere formation. (**h**) Quantification of neural sphere in hUC-MSCs-CM group and control group (*n*=6). (All data shown as mean±S.E.M., *P<0.05, ***P<0.001, scale bar, 50 *μ*m.)

**Figure 5:**
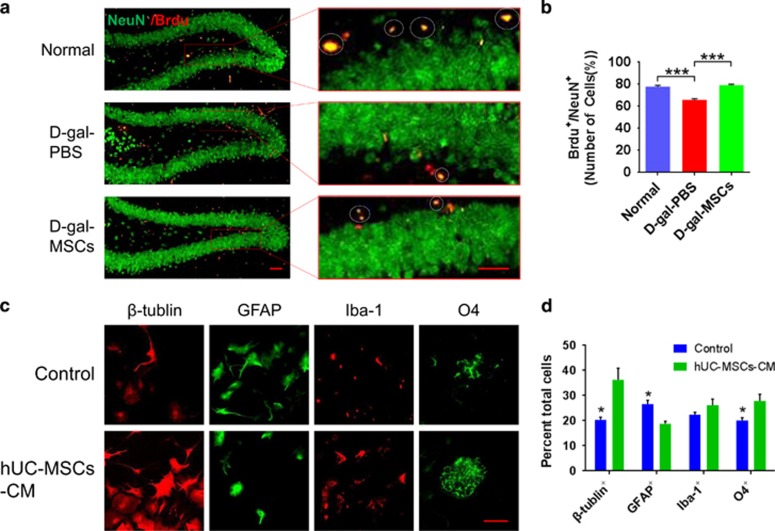
hUC-MSCs enhanced the ability of NSCs differentiation to neuron. (**a**) Representative images of hippocampal dentate gyrus showing newborn neurons in normal, d-gal-PBS and d-gal-MSCs mice. Circles in higher-magnification inserts indicate BrdU^+^/NeuN^+^ double-positive cells. (**b**) Quantification of neurogenesis in the hippocampal of normal, d-gal-PBS and d-gal-MSCs mice (*n*=4). (**c**) Immunofluorescence staining showed that the proportions of *β*-tubulin^+^, GFAP^+^, Iba1^+^ and O4^+^ cells are altered in the presence of hUC-MSC-CM group compared with control group. (**d**) Quantification of cell types in the presence and absence of hUC-MSC-CM. Compared with that in control, the proportion of *β*-tubulin^+^, Iba1^+^ and O4^+^ is increased by hUC-MSC-CM. (All data shown as mean±S.E.M., *P<0.05, ***P<0.001, scale bar, 50 *μ*m.)

In the aged brain, neural stem/progenitor cells and neurogenesis decline, leading to reduced neuroplasticity and cognitive function.^6^ Adult neurogenesis usually occurs in neurogenic niches in the subventricular zone (SVZ) and the DG of the hippocampus.^6^ To explore whether hUC-MSCs can affect the neurogenesis, we analyzed coronal SVZ sections of mice for proliferative Ki67^+^ cells and coronal hippocampal sections for SOX2^+^ stem cells. Results showed that a decrease in the number of proliferative Ki67^+^ cells in SVZ (Figures 4a and b) and SOX2^+^ stem cells in DG (Figures 4c and f) in the d-gal-PBS group, as compared with that of the normal and d-gal-MSCs groups (Figures 4d and e). Furthermore, we assessed neuronal differentiation using a long-term BrdU incorporation assay, by which mature differentiated neurons expressed both of the neuronal marker NeuN and Brdu (Figure 5a). d-gal-PBS group showed a significant decrease compared with that of the control and d-gal-MSCs group (Figures 5a and b). In addition, we confirmed that exposure of primary mouse hippocampal neural stem cells (NSCs) to hUC-MSCs-CM resulted in increased proliferation (Figures 4g and h), and neuronal differentiation (Figures 5c and d) *in vitro*. Together, these results demonstrate that hUC-MSCs activate the endogenic NSCs proliferation and differentiation into functional neurons, which is beneficial to stabilize the neural network in hippocampus.

### hUC-MSCs regulated hippocampal synaptic plasticity via ERK-CREB pathway

**Figure 6:**
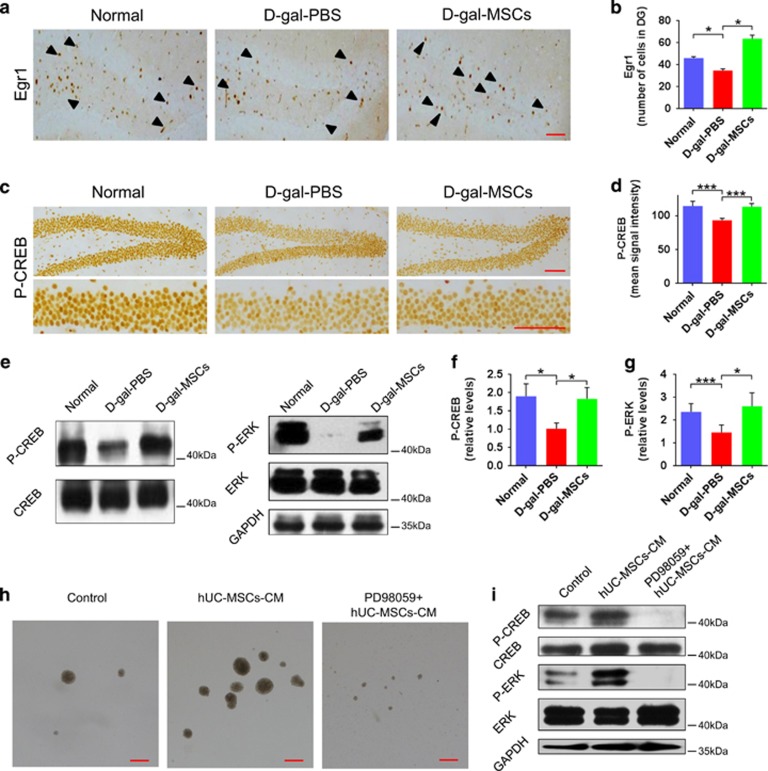
hUC-MSCs regulated hippocampal synaptic plasticity via ERK-CREB pathway. (**a**,**c**) Immunohistochemical detection of Egr1, and phosphorylated CREB (P-CREB) protein in the DG of normal, d-gal-PBS and d-gal-MSCs mice, arrowheads indicate individual cells. (**b,d**) Quantification of the immunostaining for (**b**) Egr1, and (**d**) P-CREB. Five sections per mouse were analyzed (*n*=6 mice). (**e**) Representative images and (**f**,**g**) quantification of western blotting showing the expression of P-ERK, P-CREB in the hippocampus of normal, d-gal-PBS and d-gal-MSCs mice. Representative images of the effects of hUC-MSCs-CM with and without PD98059 on (**h**) proliferation of NSCs *in vitro* and (**i**) phosphorylation of ERK and CREB. (All data are shown as the mean±S.E.M., *P<0.05, ***P<0.001, scale bars, 50 *μ*m)

To further explore how hUC-MSCs facilitate hippocampal structure and function, and enhance cognition, we then examined a subset of key proteins involving synaptic plasticity by immunohistochemistry in mice. We found that an outstanding increase of cells expressing the immediate early genes Egr1 (Figures 6a and b) and a corresponding increase in phosphorylated CREB (P-CREB) (Figures 6c and d; Supplementary Figure 4A) in the DG of d-gal-MSCs group compared with d-gal-PBS group. The activation of ERK^19^ or PKA^20^ has an important role in hippocampal synaptic plasticity and memory, and the former promotes hippocampal neurogenesis.^21^ To elucidate the mechanism by which hUC-MSCs activates Egr1 expression, two important kinases, ERK and PKA, known to mediate neuronal transcriptional events, were analyzed by western blotting. We found that the phosphorylation of ERK at Thr202/Tyr204 and CREB at Ser133 (Figures 6e-g; Supplementary Figure 4C) was upregulated in the hippocampus of d-gal-MSCs group, comparing with that of d-gal-PBS group; while there was no significant changes in phosphorylation of PKA at Thr197 and Ser338 in three groups (Supplementary Figure 2). These data demonstrate that hippocampal structure and function enhancements elicited by exposure to the secretome of hUC-MSCs are mediated, at least partially, by activation of the cyclic AMP response element binding protein (CREB) in the aged hippocampus through the activation of MAPK-ERK signaling pathway.

Meanwhile, *in vitro* experiment, we found that exposure of NSCs to hUC-MSCs-CM resulted in increased proliferation (Figure 6h) and upregulated phosphorylation of ERK at Thr202/Tyr204 and CREB at Ser133 (Figure 6i); however, pretreatment with PD98059, an inhibitor of ERK, could effectively suppress the proliferation and phosphorylation promoting effects of hUC-MSCs-CM on NSCs (Figures 6h and i). It suggested that hUC-MSCs-CM regulates the proliferation of NSCs through the ERK-CREB pathway.

## Discussion

Cognitive aging and its influence on cognitive health are matters of pressing public health importance.^3^ At present, healthy lifestyle, such as physical exercise, cognitive stimulation, avoiding excessive exposure to neurotoxins and so on, may be beneficial for cognitive aging,^22^ but it is not enough or specific to prevent cognitive aging. So in fact, new strategies of specific interventions for cognitive aging need arising pressingly.

Recently, the transplantation of specific tissue-derived mesenchymal stem cells (MSCs) has been shown to be effective in the repairment or regeneration of several tissues, such as bone, heart and lung.^23, 24, 25^ It is generally accepted that the efficacy of MSCs is based on the secretion of a wide range of substances, including growth factors, cytokines, chemokines and metabolites which are very important to regulate multiple physiological functions of the organism.^26, 27^ Compared to many other adult tissue-derived MSCs, hUC-MSCs, maintaining an earlier embryologic phase, are much younger and can secrete a wide range of multifunctional factors. So, we considered hUC-MSCs may be a preferred resource to systematically regulate the aging brain and specially interpose cognitive aging, and we had indeed certified that in aging mice. More importantly, as the ideal stem cells for universal application, hUC-MSCs can be allogeneic transplantation due to their immunological naivity and weaker response to inflammatory stimuli. Currently, hUC-MSCs are under investigation for a variety of clinical therapeutic trials, such as neurological deficits,^28^ liver diseases,^29^ immune system diseases,^30^ and several of them have completed on Phase I or II.^8^ Therefore, the application based on hUC-MSCs for intervening cognitive aging may be an advantage strategy in future.

A large number of basic and clinical trial researches have shown that the number and especially the quality of stem cells are critical for the safety and effectiveness of clinical treatment. In our study, we greatly optimized the traditional tissue block method. Then the stem cells obtained were not only significantly increased in number, but also certified by the national authority in quality (Figure 1; Table 1; Supplementary Table 1). This ensures the reliability of our research data on one hand, on the other hand, makes full preparations for the next clinical research. More importantly, our optimized method would be hopeful of improving engraftment for efficient and less costly cellular therapy for adult stem cells.

To better have the role of systematical regulation of hUC-MSCs, the preparations composition and appropriate method of injection have been analyzed and confirmed in this study. It has been reported that the secretion of a specific composition of neurotrophic factors from MSC is enhanced in the pathological conditions, such as pro-inflammatory and hypoxic stimuli.^31, 32^ MSCs transplantation is more effective than the secretion group injection. Therefore, we choose the way of direct infusion of hUC-MSCs in this study. As we known, intracranial transplantation may result in damage to the intact brain tissues. Meanwhile, by intravascular transplantation, MSCs are prone to be entrapped in the lungs, potentially increasing the risk for iatrogenic atelectasis and lethal pulmonary thromboembolism.^33, 34^ Previous researches have shown that intraperitoneal administration of MSCs to mice can confer significant lifespan and healthspan extension,^35^ alleviate neuropathology and symptoms associated with globoid cell leukodystrophy.^36^ By cell tracer testing, we also detect that hUC-MSCs have a certain time of survival *in vivo* after intraperitoneal injection, and the lipophilic tracer signal of hUC-MSCs mainly concentrate in the liver, a small amount in the heart, lung, kidney, spleen and brain (Supplementary Figure 3A). However, the human nuclear antigen (HuN) was not detectable in the mice organs (Supplementary Figure 3B). It suggested that no or very few hUC-MSCs entered the circulation after intraperitoneal transplantation, but the microvesicles (which were marked with DiI) as well as cytokines secreted from hUC-MSCs could enter into the mice circulation system and then play important roles. All these data suggested the intraperitoneal injection as one of the optimal candidate transplantations, which is much safer and can realize the systematical regulation of hUC-MSCs with the capacity of secrecting multifunctional factors. Indeed, we find that the intraperitoneal administration of hUC-MSCs effectively improve physiological structure and function of the neuron in the aged hippocampus and reverse age-related cognitive function in the brains of aged mice.

What are the underlying mechanisms that mediate the ability of hUC-MSCs to rejuvenate the physiological characteristics of cognitive aging? As previously reported, there are significant alterations in neuronal structure and function in the hippocampus and the ability of neural regeneration decreases in aging brain, all of which can lead to cognitive decline.^6, 37, 38^ Our study shows that hUC-MSCs increase the number of dendritic spines and the thickness of the postsynaptic density, and recover the structure of neural sheath, neuromicrotubes and neurofilaments in aging hippocampus (Figure 3). Moreover, hUC-MSCs can induce a significant higher magnitude of SC–CA1 LTP, which is crucial for synaptic transmission (Figures 2g-i).We further demonstrate that hUC-MSCs can activate the endogenous neural stem cells, and promote neurogenesis in aged brain, which have significant effects on maintaining the integration of neurocircuitry (Figures 4a–f and 5a). All these findings support hUC-MSCs reversing cognitive aging by strengthening the synaptic plasticity and neurogenesis in aging mice.

**Figure 7:**
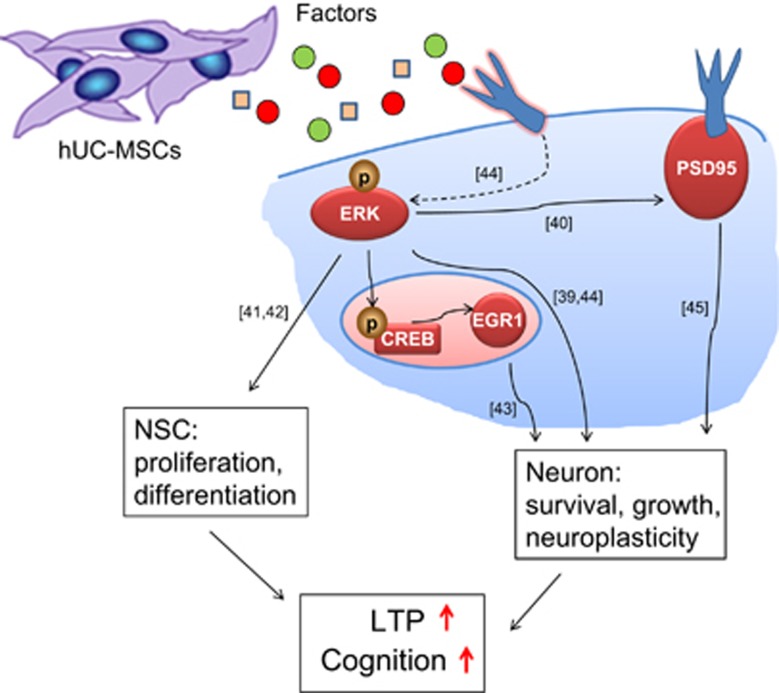
Hypothesized model for the signal pathways involved in hUC-MSCs reversing age-related cognitive aging

Recent reports implicate MAPK-ERK cascade is essential for characterized neuronal transcriptional events, and might also regulate synaptic targets to control plasticity.^19^ Here, we demonstrate that hUC-MSCs upregulate the EGR1 and promote the phosphorylation of ERK at Thr202/Tyr204 and CREB at Ser133 in the hippocampus of aging mice (Figure 6; Supplementary Figure 4). We suppose hUC-MSCs could activate the intracellular signaling cascades, MAPK-ERK pathway, which then activate several crucial effectors (Figure 7): phosphorylation of CREB at Ser133,^39^ EGR1 and PSD95,^40^ which are crucial for recovery of the structure and vitality of the neuron, synaptic plasticity and cognitive ability; activation of ERK pathway can also promote endogenic neural stem cell neurogenesis,^41, 42^ which can effectively supplement newborn neuron and maintain the neuronal network. Therefore, intracellular signaling cascades MAPK-ERK pathway activated by hUC-MSCs may have important significance for reversing age-related cognitive decline.^43, 44, 45^

To our knowledge, this is the first study to reveal that hUC-MSCs have beneficial effects on cognitive aging, and the observation that hUC-MSCs can modulate brain aging in mice presents more questions and opportunities than answers. There is still a valuable research topic that which core functional factors secreted from hUC-MSCs has important roles in hUC-MSCs-mediated recovery of cognition function. We supposed that many bioactive molecules, such as neurotrophic factors, growth factors and hormones, may play an integrated role in the progress; some of these molecules might have direct therapeutic effects for cognitive aging. We hope that further basic research will address the exciting questions that surround the origins of these factors, how they signal to the brain and why they change with age. In summary, our results demonstrate that intraperitoneal administration of hUC-MSCs can counteract cognitive aging at the synaptic plasticity, neural networks, molecular regulation, and cognitive levels in the aged brain, thus shedding light on interventions targeting the physiological cognitive decline; more importantly, it is hoped that by using such knowledge to alter basic processes involved in normal brain aging, it would become feasible to counter the cellular abnormalities that lead to neurodegeneration.

## Materials and methods

### Isolation, cultivation and identifition of hUC-MSCs

Samples of human umbilical cords were collected from Beijing 301 hospital, and stored in DMEM medium (Gibco, Grand Island, NY, USA) with penicillin-streptomycin (1:100; Gibco) at 4 °C. The isolation, cultivation and identifition of hUC-MSCs were greatly optimized based on previous description,^46^ detailed in Result.

### Condition medium collection

hUC-MSCs were seeded at initial density of 1 × 10^4^/cm^2^ in 10 cm dishes, cultured for 24 h, and the medium replaced with 8 ml of *α*-MEM for additional 48 h, conditioned medium (CM) was centrifuged (2500 rpm for 5 min) to remove cell debris and used for experiments.

### Neural stem cell cultivation, proliferation and differentiation

Neurosphere cultures were prepared from newborn rat cortex and grown in serum-free medium containing EGF and bFGF (both 20 ng/ml), For the differentiation assay, neurospheres were dissociated and plated on poly-lysine coated coverslips in the absence or presence of hUC-MSCs-CM for 3 d before analyses.^47^

To verify whether the ERK-CREB pathway is involved in the regulation of hUC-MSCs-CM (without any other additional factors) on NSCs, NSCs was pretreated with DMEM/F12 (Gibco) for 12 h. Next, NSCs was pretreated with 20 *μ*M of PD98059 (#9900, Cell Signaling Technology) for 1 h in PD98059 group. Then, the three groups were severally treated with DMEM/F12, hUC-MSCs-CM and 20 *μ*M of PD98059 in hUC-MSCs-CM for 6 h to evaluate the phosphorylation of ERK and CREB, and 3 days to proliferation observation.

### Animals

Male C57BL/6 mice, 8 weeks old were used for the study. They were housed in a conventional state under adequate temperature and humidity control with a 12 h light/12 h dark cycle and could freely access food and tap water. All animal procedures were in strict accordance with the Academy of Military Medical Sciences for the Care and Use of Laboratory Animals and were approved by the Beijing Medical Experimental Animal Care Commission. All of the experiments were conducted with an effort to minimize the number of animals used and the suffering caused by the procedures used in this study.

### Treatments

Two-month-old mice were divided into two groups: control (Normal, *n*=10) and d-gal (*n*=26)-treated group which received Subcutaneous injections of D-galactose (100mg/kg, Sigma, St. Louis, MO, USA) every day for 8 weeks. At the age of 7 months, the latter group was further classified into two subgroups (*n*=13 in each subgroup): (I) d-gal-PBS group; (II) d-gal-MSCs, they were intraperitoneally administered 5 × 10^6^ hUC-MSCs once every two weeks for 12 weeks.

Mice were injected with BrdU (50 mg/kg) and pulsed every 12 h for 3 days before sacrifised to label newborn neural cells. Mice were sacrificed and analyzed after a total of 10 months since the injections of d-galactose.

### Water maze performance

The Morris water maze apparatus was previously described.^48^ Mice were trained once a day over four consecutive days. In each trial the mouse swam until it found the platform, or after 60 s it was guided to the platform; the mouse was then placed on the platform for 15 s before being picked up. At the end of the testing period, place navigation (60 s) was performed. The spatial probe was done 5 days after navigation test. Removed the platform and recorded the crossing number of original platform position in 60 s. Data were collected and analyzed using Water Maze system (Biobserve).

### Histology and immunohistochemistry

Mice were perfused transcardially with 50 ml PBS, followed by 50 ml of 4% paraformaldehyde and their brains were removed and post-fixed overnight in 4% PFA. Each brain was embedded in paraffin and 5 *μ*m-thick coronal sections. Standard histological Hematoxylin-Eosin staining procedure was performed. Tissue sections were incubated overnight with rabbit anti-Egr1 (Cell Signaling Technology, Beverly, MA, USA) or rabbit anti—P-CREB (Ser133, 06-519, Millipore, Temecula, CA, USA) primary antibodies, and staining was revealed using biotinylated secondary antibodies and the ABC kit (Vector with diaminobenzidine (DAB; Sigma)). Individual cell numbers were quantified by Egr1, and P-CREB was quantified as the mean signal intensity using Image-Pro Plus 6.0 8 software (Media Cybernetics, Bethesda, MD, USA).

### Immunofluorescence staining and Nissl staining

Each brain was embedded in OCT. 8 *μ*m-thick coronal sections and cell cultures pre-incubated in 10% normal goat or donkey serum, Triton 0.3% in PBS for 30 min. For BrdU detection, sections were incubated for 30 min with 2 N HCl before blocking. Tissue sections or cell cultures were incubated overnight at 4 °C with the following antibodies: rat monoclonal anti-BrdU (Abcam, Cambridge, UK), rabbit polyclonal anti-Sox2 (Abcam), polyclonal rabbit anti-Ki67 (Cell Signaling Technology), mouse monoclonal anti-NeuN (Abcam), mouse monoclonal anti-*β*-Tubulin III (Millipore), mouse monoclonal anti-Iba1 (Abcam), mouse monoclonal anti-O4 (Millipore), mouse monoclonal anti-GFAP (Millipore). Secondary antibodies (1:100, Jackson ImmunoResearch Laboratories, West Grove, PA, USA). Nuclei were visualized using 2 *μ*M DAPI (Sigma). For nissl staining, washed the sections for 10 min in PBS with 0.1% Triton X-100. Apply approximately 200 *μ*l of the diluted stain (N21480, Invitrogen, Carlsbad, CA, USA) to the slide, and incubate for 20 min. Wash the sections two times for 5 min each in PBS, then washed overnight at 4 °C in PBS. Individual cell numbers were quantified by Ki67 using Volocity 6.1.1 software (Perkin Elmer, Waltham, MA, USA). In neural stem cell differentiation assays, images of the whole well (15 fields/well, 10 × magnification) were acquired using the Operetta high content automated imaging microscope.

### Golgi staining

In this study we used Golgi staining to examine the density of dendritic spines with light microscopy. The procedure followed the manufacturer's instructions of Hito Golgi–Cox OptimStain PreKit (Hitobiotec, Kingsport, TN, USA). At least three pyramidal neurons from the hippocampus CA1 region per mouse were counted. Serial focal plains of all selected dendrites were measured to ensure that all spines on tertiary branches were counted.

### Extracellular electrophysiology

Mice were sacrificed by decapitation after anesthesia using 1% pentobarbital sodium (50 mg/kg). Brain was removed carefully and immediately soaked in ice-cold incubation solution (ACSF: 1 liter H_2_O; NaCl, 6.838 g; KCl, 0.268 g; KH_2_PO_4_·2H_2_O, 0.187 g; CaCl_2_·2H_2_O, 0.3675 g; MgCl_2_·6H_2_O, 0.244 g; NaHCO_3_, 2.1 g; and glucose, 1.98 g) which had been well saturated through exposure to 95% O_2_ and 5% CO_2_ for 1 h at room temperature. Brain slices (400 *μ*m-thick) were prepared using vibrating tissue slicer (Microslicer™DTK-1000; DOSAKA), and soaked in oxygenated ACSF for 1 h at 32 °C. The hippocampal regions were resected and placed on the center on the MED-P515A probe (Alpha MED Scientific) with 64 embedded recording electrodes. After positioning of the slice on MED probe, microscopic photograph was taken. MED probe was placed on the MED connector and incubation solution oxygenated with 95% O_2_ and 5% CO_2_ was continuously infused at the rate of 2 ml/min (at 32 °C). The field excitatory postsynaptic potentials (fEPSP) were recorded using a MED64 multichannel recording system (Mobius Win7 0.5.0, WitWerx Inc.), and the data were collected from the dendritic layer of the CA1 region at a sampling rate of 10 kHz. For each slice, the baseline stimulus intensity was set at the level that elicited ~50% of the maximum fEPSP response, which was determined according to the input–output curve. Long-term potentiation (LTP) was induced by 3 trains of HFS (100 Hz for 1 s delivered 30 s apart). LTP was calculated as the mean percentage change in the amplitude of the population spike after high-frequency stimulation relative to its basal amplitude.^49^

### Western blot analysis

Mice hippocampal were dissected after sacrificed, snap frozen and lysed in RIPA buffer (50 mM Tris-HCl, pH 7.5, 150 mM NaCl, 1% NP-40, 0.5% sodium deoxycholate, 0.1% SDS), protease inhibitor cocktail tablets (Roche, Germany) and Phosphatase inhibitor cocktail tablets (Roche). Western blots were performed and analyzed as previously described.^50^ Antibodies used for Western blots were: Sox2 (ab97959, Abcam), *β*-Tubulin III (T2200, Sigma), GAPDH (#8884, Cell Signaling Technology), PSD95 (#3450, Cell Signaling Technology), rabbit Creb (04-218, Millipore), P-CREB (Ser133,06-519, Millipore), PKA (ab108385, Abcam), phospho-PKA (Ser338, ab5816, Abcam), phospho-PKA (Thr197, ab75991, Abcam), ERK 1/2 (#4695S, Cell Signaling Technology), phospho-ERK 1/2 (Thr202/Tyr204, #4376S, Cell Signaling Technology).

### Electron microscopy5

Mice were perfused with 2.5% glutaraldehyde. Hippocampal were isolated, dorsally sectioned with a vibratome at 400 *μ*m and then resectioned to 100 *μ*m. Hippocampal were post-fixed in 1% OsO4 for 1 h, dehydrated and embedded in epoxy resin. Electron micrographs (100 *μ*m^2^ CA1 and DG area at 8000X) were made of hippocampal sections with an electron microscope (Hitachi H-7650).

### Statistical analysis

All data are shown as means±S.E.M. The intensity of IHC staining, were analyzed using Image-Pro Plus 6.0 software (Media Cybernetics, Maryland, USA). Statistical analyses were performed with Prism 5.0 software (GraphPad Software, La Jolla, CA, USA). The statistical significance of the differences between two groups was determined using unpaired two-tailed Student’s *t*-test as indicated in bar graph. A value of *P*<0.05 was considered to be statistically significant.

## Figures and Tables

**Table 1:** Survey report of hUC-MSCs (Passage 5) by NIFDC

**Test items**	**Standard stipulations**	**Test results**
[Cell identification test]		
Morphology test	Adherent growth, in long spindle shape	Meet the criteria
Cell strain identification (human STR map analysis)	Being the single cell source which expresses human 16 alleles	Meet the criteria
		
Cell surface antigen		
CD73, CD90, CD105 (%)	⩾95.0	Meet the criteria
CD11b, CD19, CD34, CD45, HLA-DR (%)	⩽2.0	
Contamination test between Species	Human cells without contamination between species	Meet the criteria
**[Sterility test]**	Sterile growth	Meet the criteria
**[Mycoplasma test]**	Negative	Meet the criteria
**[Exogenous virus test-*in vitro*** **method]**	Negative	Meet the criteria
**[Exogenous virus test-*in vivo*** **method]**	Negative	Meet the criteria
[**Special human virus test**] HIV-1, HBV, HCV, HCMV, EBV, HPV; HHV-6, HHV7	Negative	Meet the criteria
[**Bovine-derived virus test**]	Negative	Meet the criteria
[**Swine-origin virus test-PPV]**	Negative	Meet the criteria
[**Retroviruses test**]	Negative	Meet the criteria
[**Immunological reaction test**]	Report results	Meet the criteria
[**Differentiation ability test**]	Report results	Meet the criteria
[**Cell activity test**]	Report results	Meet the criteria
[**Tumorigenicity test**]	Report results	Meet the criteria

Abbreviation: NIFDC, National Institutes for Food and Drug Control.

Report No: SH201700132.
