# Supplementary material for: Clinical-grade human umbilical cord-derived mesenchymal stem cells reverse cognitive aging via improving synaptic plasticity and endogenous neurogenesis
Source: Cell Death Dis. 2017 Aug 10;8(8):e2996–. doi: 10.1038/cddis.2017.316 (PMC5596535; doi:10.1038/cddis.2017.316)
Supplement: Supplementary Information [file cddis2017316x1.docx]

**Table S1. Survey report of hUC-MSCs (Passage 15) by NIFDC*.  *Report No: SH201700133***

| **Test items** | **Standard stipulations** | **Test Results** |
| --- | --- | --- |
| **[Cell identification test]** |  |  |
| Morphology test | Adherent growth, in long spindle shape | Meet the criteria |
| Cell strain identification  (human STR map analysis) | Being the single cell source which expresses human 16 alleles | Meet the criteria |
| Cell surface antigen |  |  |
| CD73, CD90, CD105 (%)  CD11b, CD19, CD34, CD45, HLA-DR (%) | ≧ 95.0  ≦ 2.0 | Meet the criteria |
| Contamination test between  species | Human cells without contamination between species | Meet the criteria |
| Karyotype analysis | The chromosome number should be 46XX or 46XY, the chromosome structure should be no missing, repeat,inversions and translocations | Meet the criteria |
| **[Sterility test]** | Sterile growth | Meet the criteria |
| **[Mycoplasma test]** | Negative | Meet the criteria |
| **[Exogenous virus test-*in vitro* method]** | Negative | Meet the criteria |
| **[Exogenous virus test-*in vivo* method]** | Negative | Meet the criteria |
| **[Retroviruses test]** | Negative | Meet the criteria |
| **[Immunological reaction test]** | Report results | Meet the criteria |
| **[Differentiation ability test]** | Report results | Meet the criteria |
| **[Tumorigenicity test]** | Report results | Meet the criteria |

***NIFDC: National Institutes for Food and Drug Control**

**Table S2. Antibodies** **used in the experiments.**

| **Name** | **Company** | **Number** | **Description** |
| --- | --- | --- | --- |
| Ki67 | Cell Signaling Technology | #9129 | Rabbit mAb |
| Sox2 | Abcam | Ab97959 | Rabbit mAb |
| Brdu | Abcam | Ab6326 | Rat mAb |
| NeuN | Abcam | Ab104225 | Rabbit mAb |
| GFAP | Cell Signaling Technology | #3670 | mouse mAb |
| Iba-1 | Abcam | ab5076 | Goat mAb |
| O4 | R&D | MAB1326 | mouse mAb |
| Tuj-1 | R&D | MAB1195 | mouse mAb |
| β-Tubulin III | Sigma | T2200 | Rabbit mAb |
| PSD95 | Cell Signaling Technology | 3450 | Rabbit mAb |
| EGR1 | Cell Signaling Technology | #4153 | Rabbit mAb |
| CREB | Millipore | 04-218 | Rabbit mAb |
| P-CREB(Ser133) | Millipore | 06-519 | Rabbit pAb |
| ERK | Cell Signaling Technology | #4695S | Rabbit mAb |
| P-ERK(Thr202/Tyr204) | Cell Signaling Technology | #4376S | Rabbit mAb |
| Anti-PKA | Abcam | Ab108385 | Rabbit mAb |
| Anti-PKA (s338) | Abcam | Ab5816 | Rabbit mAb |
| Anti-PKA (T197) | Abcam | Ab75991 | Rabbit mAb |
| GAPDH | Cell Signaling Technology | #8884 | Rabbit mAb |

**Supporting Figure Legends**

**Figure S1. hUC-MSCs protecte neuronal cell in the hippocampus. (A)** Hematoxylin and eosin staining in the dentate gyrus of the normal, D-gal-PBS, and D-gal-MSCs groups. Note that the condensation of cytoplasm is pronounced in the D-gal-PBS group, and this pattern nearly disappears in the D-gal-MSCs group. **(B)** Nissl staining in the CA1 and dentate gyrus. (GCL, granule cell layer; ML, molecular layer; and PL, polymorphic layer. Scale bar = 50μm)

**Figure S2. PKA signaling pathway analysis.** Representative images of western blotting showing the expression of P-PKA(t197), P-PKA(s338) in the hippocampal of normal, D-gal, and D-gal-MSCs mice.

**Figure S3. Tracking of the hUC-MSCs in aging mice after intraperitoneal transplantation. (A)** *Ex vivo* images showing the distribution of DiR^+^ signal in different organs as detected by the IVIS (*In Vivo* Imaging System) at day 1, day 7 and day 21 after intraperitoneal transplantation of DiR-hUC-MSCs. **(B)** Detection of DiI^+^ and HuN^+^ signal in different organs as detected by immunofluorescence at day 1, day 7 and day 21 after intraperitoneal injection of DiI-hUC-MSCs.

**Figure S4. hUC-MSCs regulated hippocampal synaptic plasticity via ERK-CREB pathway. (A)** Immunohistochemical detection of phosphorylated CREB (P-CREB) protein in the DG of normal, D-gal-PBS, and D-gal-MSCs mice after 2 months since the first injection of MSCs. **(B)** Isotype control of immunohistochemistry. **(C)** Representative images of western blotting showing the expression of P-ERK, P-CREB in the hippocampus of normal, D-gal-PBS, and D-gal-MSCs mice after 2 months since the first injection of MSCs. (Scale bars = 50 µm)

**Supporting Materials and Methods**

**Labeling and tracking of the hUC-MSCs**

After the hUC-MSCs were labeled with lipophilic tracer 1,1-dioctadecyl-3,3,3,3-tetramethylindotricarbocyanine iodide (DiR, Molecular Probes), DiR-hUC-MSCs (5×10^6^) were injected intraperitoneally to the C57/BL6 male mice (7 months old). The DiR fluorescent signal was detected in heart, liver, lung etc, when the organs were taken out and imaged directly with the IVIS (PerkinElmer IVIS) at day 1, day 7 and day 21 post transplantation. The control group received the last DiR-hUC-MSCs eluant (PBS) only.

Meanwhile, the other hUC-MSCs (5×10^6^) were labeled with DiI (Molecular Probes), and were injected intraperitoneally to the C57/BL6 male mice (7 months old). At day 1, day 7 and day 21, the organs were taken out and 8µm-thick sections were Immunofluorescence staining with anti-human nuclear antigen antibody (HuN, abcam), and then the DiI^+^ and HuN^+^ signal in different organs as detected by confocal microscopy (Nikon). The control group received the last DiI-hUC-MSCs eluant (PBS) only.

**Isotype control of immunohistochemistry**

The primary antibody was replaced with PBS, and the other steps were the same as immunohistochemistry.
