# Supplementary figures and images for: Clinical-grade human umbilical cord-derived mesenchymal stem cells reverse cognitive aging via improving synaptic plasticity and endogenous neurogenesis
Source: Cell Death Dis. 2017 Aug 10;8(8):e2996–. doi: 10.1038/cddis.2017.316 (PMC5596535; doi:10.1038/cddis.2017.316)

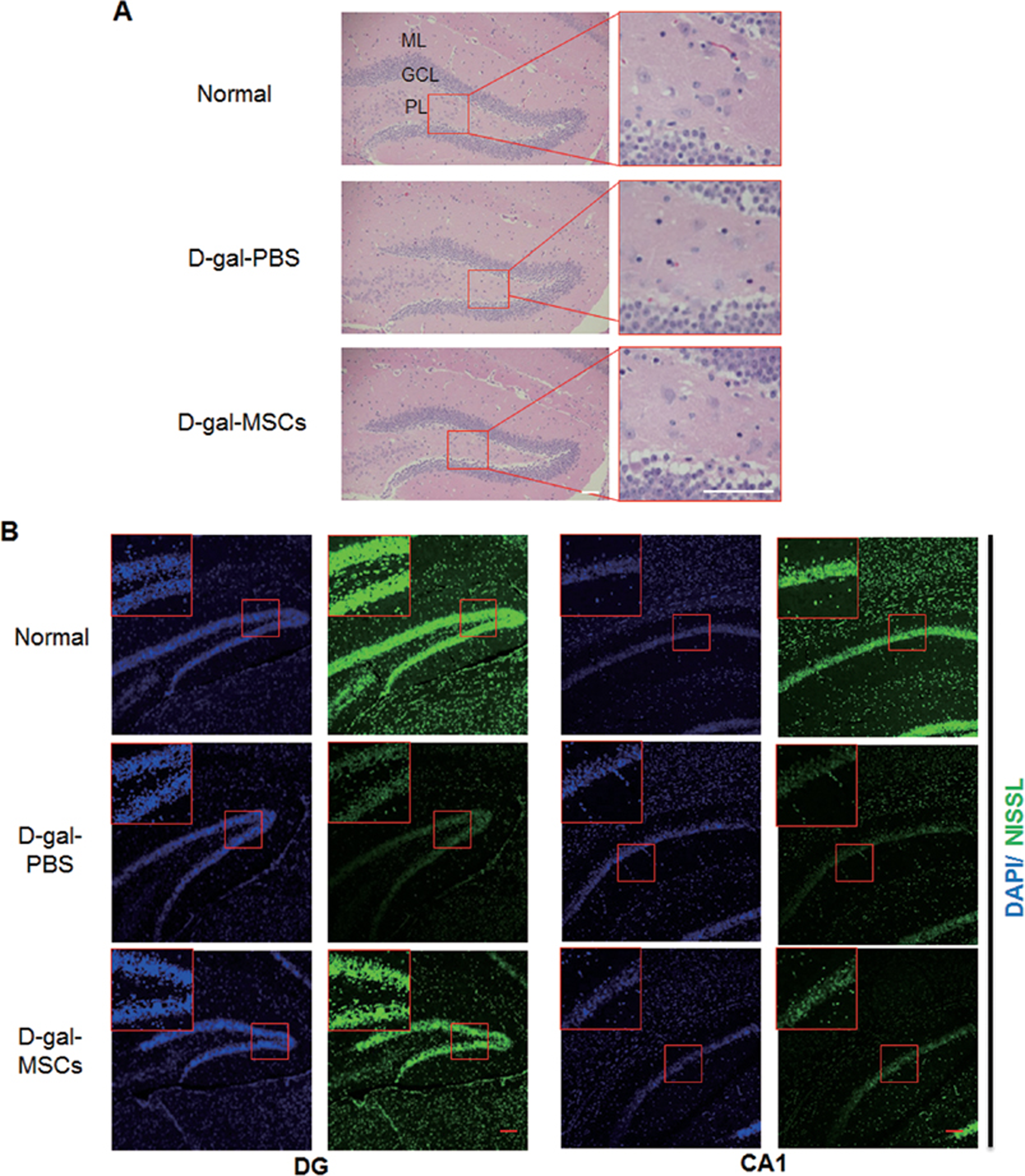

Supplement: Supplementary Figure S1 [file cddis2017316x2.tif]

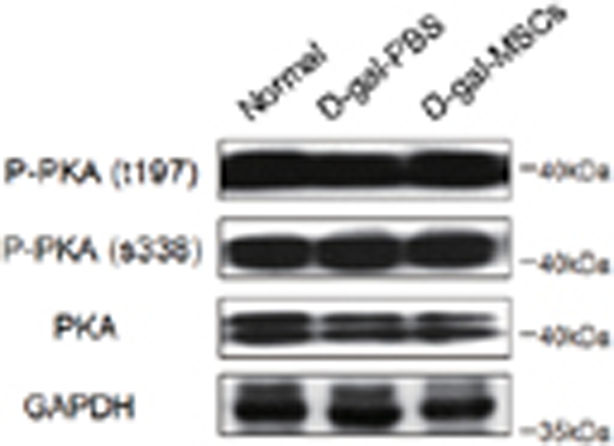

Supplement: Supplementary Figure S2 [file cddis2017316x3.tif]

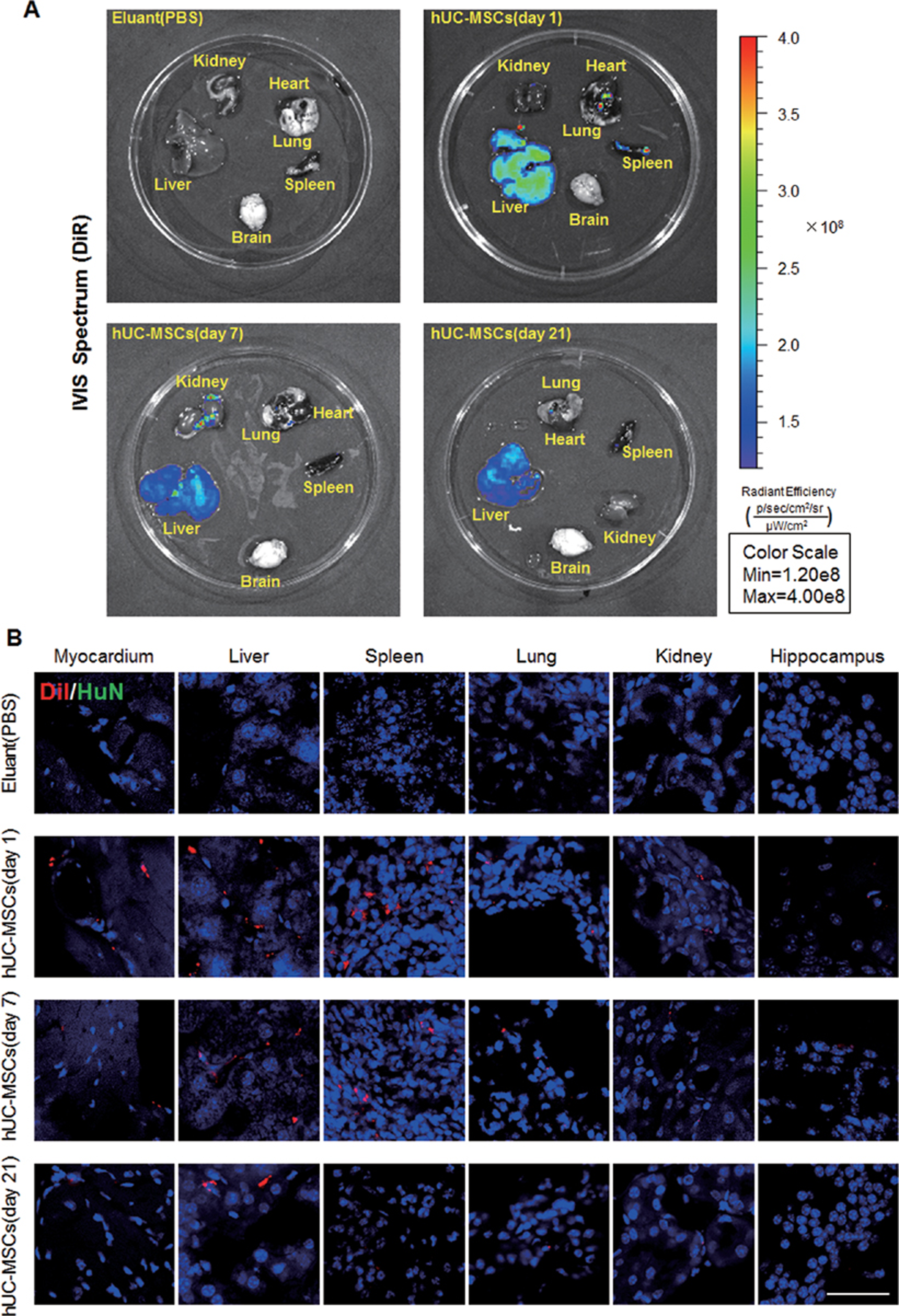

Supplement: Supplementary Figure S3 [file cddis2017316x4.tif]

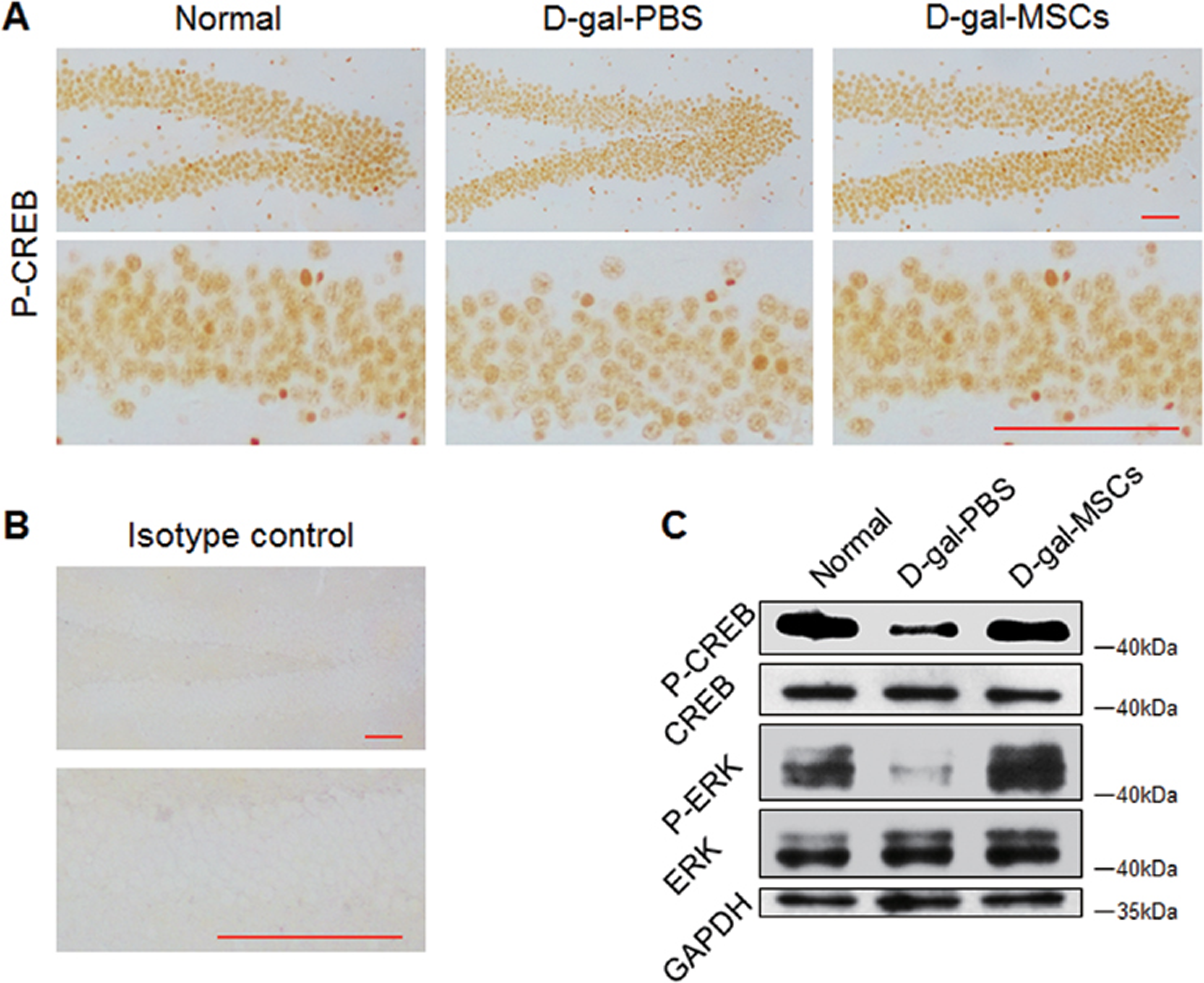

Supplement: Supplementary Figure S4 [file cddis2017316x5.tif]
